# Measuring Population Health Outcomes

**Published:** 2010-06-15

**Authors:** R. Gibson Parrish

## Abstract

An ideal population health outcome metric should reflect a population's dynamic state of physical, mental, and social well-being. Positive health outcomes include being alive; functioning well mentally, physically, and socially; and having a sense of well-being. Negative outcomes include death, loss of function, and lack of well-being. In contrast to these health outcomes, diseases and injuries are intermediate factors that influence the likelihood of achieving a state of health. On the basis of a review of outcomes metrics currently in use and the availability of data for at least some US counties, I recommend the following metrics for population health outcomes: 1) life expectancy from birth, or age-adjusted mortality rate; 2) condition-specific changes in life expectancy, or condition-specific or age-specific mortality rates; and 3) self-reported level of health, functional status, and experiential status. When reported, outcome metrics should present both the overall level of health of a population and the distribution of health among different geographic, economic, and demographic groups in the population.

*By far, the most fundamental use of summary measures of population health is to shift the centre of gravity of health policy discourse away from the inputs . . . and throughputs . . . of the health system towards health outcomes for the population. This is not to imply that the resources used and activities undertaken by national or regional health systems are unimportant; quite the contrary. But our understanding of their roles and importance is more appropriate if guided by the real "bottom line," namely their influence on population health.*

Michael C. Wolfson (1)

## Definitions and Introduction

The World Health Organization defines health as "the state of complete physical, mental, and social well-being and not merely the absence of disease or infirmity" (2). To achieve this vision of health for its members, a healthy society must establish and sustain conditions, including a healthful natural and built environment, and equitable social and economic policies and institutions, that ensure the "happiness, harmonious relations, and security of all [its] peoples" (2,3). Positive health outcomes for people include being alive; functioning well mentally, physically, and socially; and having a sense of well-being.

The level and distribution of health outcomes in populations result from a complex web of cultural, environmental, political, social, economic, behavioral, and genetic factors (Figure). In this causal web, diseases and injuries are intermediate factors, rather than outcomes, that may influence a person's health. Lung cancer, for example, has a substantial effect on physical function and lifespan, while first-degree sunburn has little effect. Health outcome metrics are standards for measuring health outcomes. Recommending a set of metrics for monitoring a population's health outcomes — as opposed to a person's health outcomes — is the objective of this essay.

**Figure:**
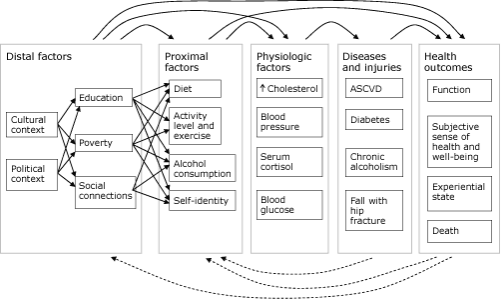
A causal web that illustrates various factors influencing health outcomes and interactions among them. Solid arrows represent potential causal relationships between factors, diseases, and outcomes. Dashed arrows represent potential feedback from outcomes and diseases on proximal and distal factors. Distal and proximal factors operate through both intermediate factors and directly on health outcomes. For example, a person's level of education can directly influence his or her subjective sense of health and level of social function and also influence intermediate factors, such as diet and exercise. Similarly, the understanding that death or loss of function may occur as the result of a person's lifestyle or social and economic factors, such as education and poverty, may influence those factors through either behavior change or changes in social or economic policy. Examples of factors, diseases, and injuries were chosen to provide a sense of the breadth of available factors. To improve readability, the relationships among proximal factors, physiologic factors, diseases and injuries, and health outcomes have been simplified. Adapted from references 4-6. Abbreviation: ASCVD, atherosclerotic cardiovascular disease.

Three approaches to measuring population health outcomes are available: 1) aggregating health outcome measurements made on people into summary statistics, such as population averages or medians; 2) assessing the distribution of individual health outcome measures in a population and among specific population subgroups; and 3) measuring the function and well-being of the population or society itself, as opposed to individual members. According to the definition of a healthy population, the third approach is the most appropriate because it focuses on how well the population produces societal-level conditions that optimally sustain the health of all people. These societal-level conditions, although not yet fully characterized or understood, most likely include an equitable distribution of power, opportunity, and resources among a population's members; social connections and interactions built on norms of reciprocity and trustworthiness (3); and environmental policies and practices that sustain the quality of the population's land, water, air, native vegetation, and animal life. These societal-level conditions may be viewed as social, economic, political, and environmental determinants of health, rather than as health outcomes, and as such are addressed by other articles in this issue of *Preventing Chronic Disease*. I focus on approaches to assessing population health outcomes in which measures of population health are constructed from the aggregation of individual-level health measures, such as mortality, functional status, and self-perceived health.

## Basic Outcome Metrics for Population Health

### Measures of mortality, life expectancy, and premature death

**Box 1.** Examples of Population Health Outcome Metrics Based on Mortality or Life Expectancy

**Mortality**
Crude mortality rate
Age-adjusted mortality rates (AAMR)
Age-specific mortality rate
Neonatal (<28 d)
Infant (<1 y) (infant deaths per 1,000 live births)
Under 5 y
Adult (15-60 y)
Other characteristic-specific mortality rates
State- or county-specific
Sex-specific
Race-specific
Condition-specific mortality rates and similar measures
Disease-specific mortality rate
Injury-specific mortality rate
Leading causes of death
Smoking-attributable mortality (number of deaths)
Maternal mortality ratio
Occupational class-specific mortality rate
**Life expectancy**
Life expectancy at birth
Life expectancy at age 65 y
**Premature mortality**
Years of potential life lost
Premature mortality rate
**Summary measures of population health**
Health-adjusted life expectancy at birth (y)
Quality-adjusted life expectancy
Years of healthy life
Healthy life years
Disability-adjusted life years
Quality-adjusted life years
**Inequality measures**
Geographic variation in AAMR among counties in a state (standard deviation of county AAMR/state AAMR)
Mortality rate stratified by sex, ethnicity, income, education level, social class, or wealth
Life expectancy stratified by sex, ethnicity, income, education level, social class, or wealth

**Box 2.** Examples of Population Health Outcome Metrics Based on Subjective (Self-Perceived) Health State, Psychological State, or Ability to Function^a^

**Health state**
Percentage of adults who report fair or poor health
Percentage of children reported by their parents to be in fair or poor health
Mean number of physically or mentally unhealthy days in the past 30 days (adult self-report)
Mean number of mentally unhealthy days in the past 30 days (adult self-report)
Mean number of physically unhealthy days in the past 30 days (adult self-report)
**Experiential and psychological state**
Percentage of adults with serious psychological distress (score ≥13 on the K6 scale)
Percentage of adults who report joint pain during the past 30 days (adult self-report)
Percentage of adults who are satisfied with their lives
**Ability to function**
Percentage of adults who report a disability (for example, limitations of vision or hearing, cognitive impairment, lack of mobility)
Mean number of days in the past 30 days with limited activity due to poor mental or physical health (adult self-report)

a Categories adapted from reference 9.

People and societies value life and health, although the relative value placed on long life versus well-being during life varies. Mortality and life expectancy are 2 basic measures of population health (Box 1).

The number of deaths that occur in a population during a period of time (usually 1 year) divided by the size of the population is the population's crude mortality. Because age is such a strong predictor of death and the age distributions of members of different populations vary, a population's mortality rate is commonly adjusted by using a standard age distribution to produce an age-adjusted mortality rate. The age-adjusted mortality rate allows comparison of mortality across different populations. One may also calculate mortality rate for a group in a population on the basis of a specific characteristic, such as age, sex, or geographic area, to yield a characteristic-specific mortality rate. Another method of assessing the effect of mortality on a population is to calculate the life expectancy of its members. Typically, this is calculated as the life expectancy at birth, although it may be calculated as the remaining life expectancy for any given age. Measures of premature death, including years of potential life lost and the premature mortality rate, quantify mortality among people younger than a particular age, typically 65 or 75 years.

Although these measures provide information about mortality and longevity, they provide no information about the contribution of specific diseases, injuries, and underlying conditions (for example, water quality, poverty, social isolation, and diet) to death, for which actions might be taken to prolong life. For this reason, disease-specific mortality rates are frequently used to illustrate the contribution of specific diseases to population mortality. Recent work extends this concept and proposes methods and measures for estimating the contributions of more fundamental causes to mortality, such as the distal and proximal factors exemplified in the causal web of the Figure (5,7,8).

### Measures of health, function, and subjective well-being

Societies and their members typically value health both subjectively (freedom from pain and suffering, joy, happiness, sense of self-worth and value to others) and objectively (ability to perform physical, mental, and social tasks) (Box 2). Measuring health in a standardized way that allows comparisons among people, countries, and cultures and over time is challenging. Various approaches, some of which have proved controversial, have been developed and used in the past 40 years. They include methods to assess and classify the health, function, and disability of members of a population, for example, the International Classification of Functioning, Disability, and Health (10), and methods to estimate the overall health of populations.

Measurements of self-perceived or "self-rated" health, functional status, and experiential state typically rely on population health surveys, such as the National Health Interview Survey (NHIS) and the Behavioral Risk Factor Surveillance System (BRFSS) in the United States, the European Union's Statistics on Income and Living Conditions, and the World Health Organization's World Health Survey. Care must be taken, however, when comparing metrics derived from different surveys: the nature and wording of questions and the time period covered may differ. Furthermore, the interpretation of health categories, such as "good" and "poor," may vary culturally among countries or even among different populations in a country. The authors of a recent study of 4 US national surveys even questioned whether self-rated health is a suitable measure for tracking population health over time because of inconsistencies in self-ratings over time among surveys and certain population subgroups (11).

Health-related quality of life (HRQL) indices are also used to quantify health and to analyze cost-effectiveness. These indices are based on interviewer- or self-administered questionnaires that address various health dimensions or domains, such as mobility, ability to perform certain activities, emotional state, sensory function, cognition, social function, and freedom from pain. Six such indices, several of which are proprietary, are used in the United States: the EuroQol EQ-5D; the Health Utilities Index Mark 2 and Mark 3; the Quality of Well-Being Scale, self-administered form; the SF-6D; and the HALex (12). More detailed descriptions of these indices are available (9,12). The Centers for Disease Control and Prevention has also developed HRQL measures that are used in BRFSS and the National Health and Nutrition Examination Survey (NHANES); these measures were recently validated against the SF-36v2 (13,14).

Although not direct measures of health and well-being, the incidence or prevalence of specific diseases and rates for accessing and using health care are frequently used as surrogates for disability, loss of function, or lack of well-being. Ascertaining the incidence and prevalence of disease may be accomplished through the use of disease registries, health records, and population surveys.

### Summary measures of population health

Summary measures of population health have been developed in the past 40 years as an alternative to or extension of the basic metrics described above. The purpose of these summary measures is to "combine information on mortality and nonfatal health outcomes to represent the health of a particular population as a single numerical index" (15). These summary measures are based on reductions in life expectancy to account for disability or other measures of poor health; they provide estimates of either the expected number of future years of healthy life at a given age or the number of years that chronic disease and disability subtract from a healthy life.

In 1971, Sullivan described techniques for calculating 2 summary health indices — life expectancy free of disability and disability expectancy — by combining mortality rates from period life tables and survey-based disability rates (16). Subsequent work has produced other summary population health measures, including health-adjusted life expectancy, quality-adjusted life expectancy, years of healthy life, healthy life years (also known as disability-free life expectancy), disability-adjusted life years, and quality-adjusted life years. These measures vary by whether they use the actual or an idealized life expectancy for the population; whether they value all years of life and disability equally or discount certain years, such as childhood and old age; whether they are expressed as an adjusted life expectancy or as a sum of the years of disability for the entire population; and how they estimate the population's health, prevalence of chronic disease, or prevalence of disability. Estimates of population health and disability are typically derived from either expert judgment in conjunction with published literature or survey data — both population and convenience samples have been used — on function, self-perceived health, and psychological or sensory distress. Along with continuing debate about methodologic issues, ethical concerns about the use of summary measures and the way in which they value life have been raised (15,17,18). Several excellent reviews on summary measures of population health and these issues are available (9,15,17,18).

### Measures of the distribution of health in a population

Measures of the distribution of health in and among populations are as relevant as measures of the level of health in and among populations (15). Understanding the distribution of health can focus attention and action on specific health determinants and population groups to reduce inequalities in health and improve the overall level of health. Although the distribution of health outcomes could be assessed on any measurable geographic, demographic, social, or economic characteristic, some researchers argue that health inequalities should be assessed by using specific social and economic characteristics that have historically determined social status (for example, wealth, ethnicity, sex, educational attainment) (19). Others suggest that this viewpoint excludes potentially relevant determinants of health (20). Metrics to assess the distribution of outcomes include measures of inequality (Gini index), measures of association (rate ratio), measures of impact (population-attributable proportion), and measures based on ranking (concentration index) (21,22).

## Attributes of a Good Health Outcome Metric

Several groups have proposed criteria for assessing and selecting specific health indicators (Table 1). Their criteria include the need for the indicators to 1) further the goals of their organization, 2) be valid and reliable, 3) be easily understood by people who use them, 4) be measurable over time, 5) be measurable for specific geographically or demographically defined populations, 6) be measurable with available data sources, and 7) be sensitive to changes in factors that influence them, such as socioeconomic or environmental conditions or public policies (23-25).

## Current Metrics for Population Health Outcomes

In 2008, Wold reviewed 35 sets of health indicators in use (26). Although not an exhaustive list, these 35 sets provide a representative view of health indicators and their intended uses, which include presenting a picture of the health of a place, stimulating action to improve health, and tracking progress toward meeting objectives (Table 2). No set of indicators is explicitly used as a guide to financially reward improvement in health outcomes.

Wold grouped the indicator sets into 4 overall categories: general health (14 sets), quality of life (5 sets), health systems performance (11 sets), and "other" (5 sets). She further divided the general health category into national (7 sets) and state and local (7 sets). These 35 indicator sets contain various health measures, only a few of which are outcome measures. Frequently used outcome indicators are infant mortality rate, condition-specific mortality rate, age-adjusted mortality rate, years of potential life lost, life expectancy at birth, leading causes of death, and percentage of adults who report fair or poor health.

## Data and Analytical Issues for Population Health Outcome Metrics

### Available data sources

The principal sources of data available for US population health outcomes are mortality data derived from death certificates and data on subjective health status, functional status, and experiential state derived from population health surveys. The National Vital Statistics System (NVSS) collects and compiles data on births and deaths from all registration districts (most commonly states) in the United States. The most commonly used surveys are NHIS, BRFSS, NHANES, and the National Survey on Drug Use and Health (NSDUH). Several states conduct city- or county-level risk factor surveys by using BRFSS methods and questions, and an increasing number of cities and counties now conduct their own surveys based on or derived from BRFSS. A few states and local areas (Wisconsin and New York City, for example) conduct surveys based on NHIS or NHANES methods to provide state or local estimates of health outcomes and determinants.

### Geographic units of analysis

Mortality data are available for states and counties. Some states geocode their vital statistics data and provide data — usually through a Web-based data query and mapping tool — for zip codes, census tracts, or locally defined areas. BRFSS provides state-level estimates and estimates for selected metropolitan statistical areas with 500 or more respondents. Several states, including Florida, North Dakota, Washington, and Wisconsin, conduct their own county-level BRFSS to produce estimates for at least some of their counties. NSDUH provides national and state estimates. NHIS and NHANES only provide national estimates.

### Validity and precision of the measures

The validity and precision of mortality data — at least the number of people who die in a given time period in a given place — are high, as death registration is virtually complete in the United States. Condition-specific mortality data may be less valid because of errors in determining and coding the cause of death.

The designs of NHIS and NHANES to ensure that their samples are representative of their target populations and their high response rates (75%-90%) are indicators of high validity. Precision of estimates is related to sample size and the amount of variation of the characteristic being estimated in the target population. The size of the NHIS sample is sufficient to provide national estimates for the total population with relative standard errors of 1% to 3%, although relative standard errors of estimates for small subgroups may be as high as 10% to 30%. To provide more precision, NHIS oversamples some population subgroups. Estimates may be obtained for most states by combining data collected in several years.

Response rates for BRFSS, a state-based telephone survey, are considerably lower than for NHIS and NHANES. For example, state response rates for the 2008 survey ranged from 20% (Connecticut) to 58% (Utah), and the median was 34% (35).

### Measuring trends

NVSS, NHIS, BRFSS, and NSDUH provide data annually, and NHANES provides data every 2 years. National trends can be measured by using any of these data sources, state trends can be measured by using NVSS and BRFSS, and county trends can be measured by using NVSS.

Annual trends in crude and age-adjusted mortality rate and in life expectancy since the mid-1900s are available for the United States at the national, state, and county levels. See, for example, an analysis of trends in county-level mortality (36), life expectancy at birth by race and sex from 1900 through 2005 (37), and average annual age-adjusted mortality by race, Hispanic origin, and state for 1979 through 1981, 1989 through 1991, and 2003 through 2005 (37). Trend data on mortality are also available for selected causes of death (37).

Trends in HRQL, assessed by using CDC's HRQOL-4 measures derived from BRFSS, are available for the United States and for each state from 1993 through 2008, the most recent year for which BRFSS data are available (13). CDC is generating county-level estimates for the following 3 CDC HRQOL-4 measures for 2001 through 2007 for the MATCH (Mobilizing Action Toward Community Health) county rankings by using BRFSS data: percentage who report fair or poor health, physically unhealthy days in the past 30 days, and mentally unhealthy days in the past 30 days. Neither national-, state-, nor county-level population data are available for the other HRQL indices. Their use has typically been in the clinical or research setting for assessing medical or surgical therapies. The Health Utilities Index has been used in Canada for 4 major population health surveys. Although many studies document the validity of various HRQL indices, fewer studies document their reliability or responsiveness to change over time.

### Measuring inequalities in health

Several characteristics are available from NVSS and each of the surveys for measuring the dependence of population health on social and economic factors (Table 3). All systems provide these 5 characteristics for analysis: age, education level, ethnicity, race, and sex. Because of the limited availability of data for smaller geographic units, none of the systems can measure inequalities in health at the county level, except NVSS.

## Recommendations

"No single measure can capture the health of the nation" (24). On the basis of this review of existing health outcome metrics and data available for counties, I recommend the following metrics for population health outcomes at the county level.

### Life expectancy from birth or age-adjusted mortality rate

This metric mirrors a relevant outcome, data are readily available to assess temporal trends and geographic and demographic variation, and mortality is amenable to population health interventions, although changes in the mortality metric may take years to appear. Life expectancy has the advantage of being more easily communicated to, and understood by, the public than mortality rates.

### Condition-specific changes in life expectancy or condition- or age-specific mortality rate

This metric has the advantages of the overall mortality metric, as above, and allows public health programs to monitor the effect of specific interventions on more specific outcomes. An example might be monitoring increases in life expectancy or reductions in motor vehicle injury-related mortality resulting from efforts to modify driver behavior and to make roads and vehicles safer.

The conditions should be selected on the basis of local needs assessments (for example, conditions that dramatically affect mortality that could be addressed by local population health programs or other interventions). Alternatively, if states or counties needed to be compared directly, a fixed set of conditions could be selected, similar to conditions that the Institute of Medicine recommended for the State of the USA indicators (infant mortality and injury-related mortality).

### Self-perceived level of health, functional status, or experiential state

This metric reflects the population's state of health and functional level and might provide a more immediate measure of the effect of interventions than the mortality metrics. Age-, sex-, and race-specific versions of the metric could provide at least some population specificity, which might be useful in monitoring the effect of interventions.

Although many of the HRQL instruments already in general use would work well for this metric, most of the instruments are proprietary, and state- and county-level data are not available from any of them. CDC's HRQOL-4 is probably the most viable option for this measure, as it is not proprietary and state-level data have been available since 1993. By using moving averages or other methods of aggregating data, county-level trend estimates could be developed even for small counties. Although data from CDC's HRQOL-4 are readily available, a more robust measure of HRQL, with specific questions about activity limitation, functional status, and experiential state, should be explored and adopted in the future (38). The CDC HRQOL-14, other HRQL indices described above, and work by Statistics Canada and REVES (Réseau Espérance de Vie en Santé, http://reves.site.ined.fr/en/home/about_reves) should be considered for this role.

### Distribution of population health outcomes

Metrics that provide only the average level of health in a population may mask inequalities in the distribution of health, with policy and programmatic implications. Metrics that provide information on the distribution of health are another component of a complete picture of population health (1,15). Such metrics would measure the inequalities in health among different geographic, economic, and demographic populations.

One geographically based metric is the rate difference between the highest and lowest county life expectancies or age-adjusted mortality rates in a state. America's Health Rankings introduced a measure in 2008 on the variation in mortality among counties in each state (27). A demographically based metric might be the difference between the highest and lowest sex- and race-specific life expectancies or age-adjusted mortality rates in a state. An economically based metric might be the difference in life expectancies or age-adjusted mortality rates between the highest and lowest income deciles in a state.

### An optional summary measure of population health

Summary measures of population health, which combine information on death and nonfatal health outcomes, have the advantage of simplicity and parsimony and may be easier to communicate to the public and track over time than the series of basic measures previously recommended. If a summary measure is desirable, the health-adjusted life expectancy and healthy life years are good choices because they are based on life expectancy and use a population-based measure of HRQL, rather than an expert judgment-based measure.

## Figures and Tables

**Table 1:** Criteria Used to Select Health-Related Indicators by 2 Institute Of Medicine Committees and Criteria Proposed to Select Global Health Indicators

**Criteria^a^ for Selecting an Indicator**	Leading Health Indicators (23)	State of the USA Indicators (24)	Global Health Indicators (25)
Indicator is well-defined.			X
Indicator is worthwhile or important.	X	X	
Indicator is valid and reliable.	X	X	X
Indicator can be understood by people who need to act.	X		X
Indicator galvanizes action.	X		X
Action can improve the indicator.	X		
Measuring the indicator over time reflects effect of action.	X		
Measuring the indicator is feasible.			X
Data for the indicator are available for various geographic levels (local, national) and population subgroups.	X	X	X
Indicator is sensitive to changes in other societal domains (socioeconomic or environmental conditions or public policies).		X	

a The criteria for selecting indicators were compiled from the 3 reports cited. An "X" indicates that a report proposed using this criterion for selecting indicators.

**Table 2:** Stated Purposes of 9 Health Indicator Sets^a^

**Indicator Set**	Purpose
America's Health Rankings (27)	To stimulate action by people, communities, public health professionals, health industry employees, and public administration and health officials to improve the health of the population of the United States
Boston Indicators Project (28)	To democratize access to information, foster informed public discourse, track progress on shared civic goals, and report on change in 10 sectors
Community Health Status Indictors (29)	To provide an overview of key health indicators for local communities and to encourage dialogue about actions that can be taken to improve a community's health
Georgia Health Equity Initiative (30)	To look holistically at the major factors that influence differences in health status and their relationship to racial and ethnic characteristics
*Healthy People 2010* Leading Health Indicators (31)	To define health objectives for the United States and track progress toward meeting them
Institute of Medicine, State of the USA Health Indicators (24)	To help Americans become more informed and, therefore, active participants in focusing public debate on important issues . . . To provide the most reliable and objective facts about the state of the United States and to serve as a tool for Americans to track the progress made on a broad range of issues, such as education, health, and the environment
Los Angeles County, Key Indicators of Health (32)	To monitor key health conditions and to engage a broad community of stakeholders in health improvement work
Robert Wood Johnson Foundation Commission to Build a Healthier America (33)	To raise visibility of the many factors that influence health, examine innovative interventions that are making a difference at the local level and in the private sector, and identify specific, feasible steps to improve Americans' health
Wisconsin County Health Rankings (34)	To summarize the current health of the counties as well as the distribution of key factors that determine future health . . . To encourage all community stakeholders to work with health departments and health care providers . . . to improve Wisconsin's health

a Eight of these sets were selected from the 35 indicator sets identified and reviewed by Wold in 2008 (26) for the Institute of Medicine's State of the USA Committee. The ninth indicator set was developed by the Institute of Medicine's State of the USA Committee. The criteria used for selecting the indicator sets displayed in this table from the 36 candidate indicator sets were that the indicator set contained both health outcome indicators and a specific stated purpose.

**Table 3:** Characteristics for Which Inequalities in Health Can Be Measured by Using 1 State Survey (BRFSS), Data from 2 National Surveys (NHIS, NSDUH), and NVSS Mortality Data

**Characteristic**	BRFSS	NHIS	NSDUH	NVSS
Age	X	X	X	X
Citizenship		X		
Education level	X	X	X	X
Employment status	X	X	X	
Ethnicity	X	X	X	X
Geographic region			X	
Income	X	X		
Insurance status		X		
Marital status	X			X
National origin				X
Place of birth		X		
Place of residence	X		X	X
Race	X	X	X	X
Sex	X	X	X	X

Abbreviations: BRFSS, Behavioral Risk Factor Surveillance System; NHIS, National Health Interview Survey; NSDUH, National Survey on Drug Use and Health; NVSS, National Vital Statistics System.
